# The Value of Contrast-Enhanced Ultrasound versus Doppler Ultrasound in Grading Renal Artery Stenosis

**DOI:** 10.1155/2020/7145728

**Published:** 2020-09-08

**Authors:** Yanhua Cui, Quanbin Zhang, Jiping Yan, Ji Wu

**Affiliations:** ^1^Department of ultrasonic medicine, First Affiliated Hospital of Guangxi Medical University, Nanning 530021, China; ^2^Department of ultrasound, Shanxi Provincial People's Hospital, Taiyuan 030012, China; ^3^Department of ultrasound, Sixth Affiliated Hospital of Shanxi Medical University, Taiyuan 030008, China

## Abstract

**Objective:**

This study is aimed at exploring the accuracy of contrast-enhanced ultrasound (CEUS) in grading renal artery stenosis.

**Methods:**

122 renal arteries with suspected renal artery stenosis were selected. DSA, DUS, and CEUS were performed for all patients with suspected renal artery stenosis in the research. DSA was selected as the gold standard. The sensitivity, specificity, accuracy, positive predictive value (PPV), and negative predictive value (NPV) of CEUS or Doppler ultrasound (DUS) in the diagnosis of renal artery stenosis were analyzed. The consistency between CEUS and digital subtraction angiography (DSA) was compared. The accuracy of DUS or CEUS in grading renal artery stenosis was assessed by the area under the receiver operating characteristic (ROC) curves and compared between groups.

**Results:**

The sensitivity, specificity, accuracy, positive predictive value, and negative predictive value of CEUS in the diagnosis of renal artery stenosis were 88.9%, 87.8%, 88.5%, 93.5%, and 80.0%, respectively. There was no significant difference in grading renal artery stenosis between CEUS and DSA (*X*^2^ = 0.643, *P* = 0.424). 77 of the 122 renal arteries were diagnosed with the stenosis rate more than 30% by CEUS. Compared with the results of DSA, the kappa value of CEUS was 0.749 (*P* < 0.05).

**Conclusion:**

CEUS is accurate in grading renal artery stenosis, and it may represent the method of choice in diagnosing renal artery stenosis.

## 1. Introduction

Renal artery stenosis elicits complex biological responses, and it often develops progressively [1, 2]. Serious health complications such as secondary hypertension, stroke, myocardial infarction, renal failure, and even death can be caused by renal artery stenosis [3–5]. Atherosclerosis is largely thought to be the most common reason of the disease [6, 7]. The stenosis of renal artery also may occur in patients with fibromuscular dysplasia, Takayasu arteritis, neurofibromatosis, embolism, and so on [8–11]. In most patients who are not properly treated at the initial stage, blood flow can be restored after renal artery dilation, but the renal function usually fails to improve [12]. Early diagnosis of renal artery stenosis may play an important role in reversing resistant hypertension and may be helpful in terms of the protection of renal function [13–15]. The risk for cardiovascular and renal complications may be significantly reduced by proper treatment at the initial stage [16]. At present, DSA remains the gold standard for the diagnosis of renal artery stenosis [17, 18]. However, DSA is an invasive and radiological method. And the reliance on nephrotoxic contrast agents also limits the use of DSA. Therefore, it is usually not the first choice in clinical practice. Although the specificity and sensitivity of renal artery enhanced computed tomography angiography (CTA) or magnetic resonance angiography (MRA) are good [19]. There are radiation hazards in CTA examination and nephrotoxicity in CTA or MRA contrast agents [20]. The sensitivity of MRA may be affected due to the technical restrictions and artifacts [13]. The cost of CTA or MRA is high and the operation is also complicated. Especially in patients with renal artery stenosis, multiple follow-up reviews are needed to determine the prognosis and recovery after treatment. Therefore, the clinical application of CTA and MRA is limited in some particular cases. Although DUS has been widely used as an initial imaging modality for the diagnosis of renal artery stenosis [21, 22], factors such as depth, obesity, complex anatomy, bowl gas, and operator dependent may limit the use of the Doppler ultrasound [23]. More recently, the use of CEUS in the detection of renal artery stenosis has raised the attention of many researchers [24, 25]. CEUS has the advantages of noninvasion, nonradiation, cost effective, and easy operation. Renal toxicity and allergic reaction may be reduced by using this imaging technique [17]. However, the value of CEUS in grading renal artery stenosis is still uncertain and it needs to be further demonstrated. In this study, we investigated the accuracy of CEUS and DUS in grading renal artery stenosis. DSA was selected as the gold standard. CEUS and DUS imaging were applied to explore the character of renal artery stenosis. The role of CEUS in grading renal artery stenosis was evaluated in the research.

## 2. Materials and Methods

### 2.1. Subjects

**Figure 1:**
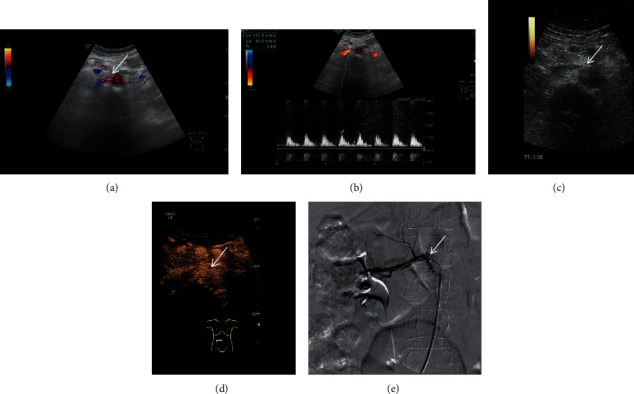
DUS images, CEUS images, and their corresponding DSA images of grade I (stenosis rate: 30%-49%). (a) Color Doppler ultrasound was used to show the long axis section of renal artery. (b) The peak systolic velocity at the stenosed segment of the renal artery was obtained by pulse Doppler ultrasound. (c) Two-dimensional grayscale ultrasound cannot clearly show renal artery stenosis. (d) Contrast-enhanced ultrasound can increase the visualization of renal artery and can be used for the display of grade I renal artery stenosis. (e) Digital subtraction angiography confirms the result of contrast-enhanced ultrasound and shows the diameter of the main renal artery decreased at the stenosed segment in the patient with renal artery stenosis.

**Figure 2:**
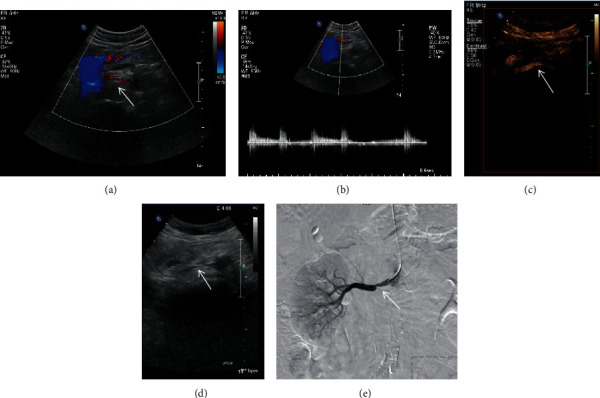
DUS images, CEUS images, and their corresponding DSA images of grade II (stenosis rate: 50%-69%). (a) Color Doppler ultrasound was used to show the long axis section of renal artery. (b) The peak systolic velocity at the stenosed segment of the renal artery was obtained by the pulse Doppler ultrasound. (c) Contrast-enhanced ultrasound can increase the visualization of renal artery and can be used for the display of grade II renal artery stenosis. (d) Two-dimensional grayscale ultrasound cannot clearly show renal artery stenosis. (e) Digital subtraction angiography confirms the result of contrast-enhanced ultrasound and shows the diameter of the main renal artery decreased at the stenosed segment in the patient with renal artery stenosis.

**Figure 3:**
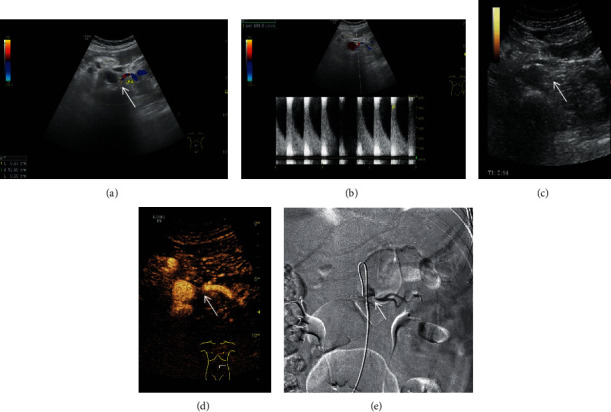
DUS images, CEUS images, and their corresponding DSA images of grade III (stenosis rate: 70%-99%). (a) Color Doppler ultrasound was used to show the long axis section of renal artery. (b) The peak systolic velocity at the stenosed segment of the renal artery was obtained by the pulse Doppler ultrasound. (c) Two-dimensional grayscale ultrasound cannot clearly show renal artery stenosis. (d) Contrast-enhanced ultrasound can increase the visualization of renal artery and can be used for the display of grade III renal artery stenosis. (e) Digital subtraction angiography confirms the result of contrast-enhanced ultrasound and shows the diameter of the main renal artery decreased at the stenosed segment in the patient with renal artery stenosis.

A retrospective research was performed from April 2015 to March 2020. A total of 122 renal arteries of 63 patients with the diagnosis of suspected renal artery stenosis were included in the research. There were 30 males and 33 females, with an average age of 57.3 ± 6.7 years in the research. All of the patients were collected in the First Affiliated Hospital of Guangxi Medical University, Shanxi Provincial People's Hospital, and the Sixth Affiliated Hospital of Shanxi Medical University. The GE Logic E9 or Philips iU22 ultrasound machine was used for CEUS and DUS examinations; both of them have a convex array probe, and the probe frequency is 3.5-5 MHz. DSA was selected as the gold standard for the diagnosis of renal artery stenosis, and it was performed for all patients with suspected renal artery stenosis in the research. The diameter of the main renal artery decreased significantly at the stenosed segment in patients with renal artery stenosis on DSA. The criteria of suspected renal artery stenosis can be determined by finding a parvus-tardus waveform, systolic acceleration time prolonged (>80 ms), peak velocity of blood flow increased (>80 m/s), ratio of renal artery flow velocity to aortic flow velocity increased (>3), and resistive index difference (≥5%) at the stenosed region of the renal artery by DUS examinations [24, 26, 27]. The results of DUS and CEUS with different grades of renal artery stenosis were compared with the gold standard DSA. In this study, DUS images, CEUS images, and their corresponding DSA images of different grades of renal arteries stenosis were obtained (Figures 1–3). Patients with nephrectomy, renal tuberculosis, renal aneurysm, and anomalous origin of the renal artery were excluded from this study.

This study was approved by the ethics committee of Shanxi Provincial People's Hospital. All participants signed an informed consent. All experimentation was conducted in conformity with ethical and humane principles of research.

### 2.2. DUS Examinations

All of the patients took the supine position or lateral position. They kept fasting for more than 6 hours. DUS was used to show the long axis section of main renal artery. The course of the main renal artery was observed. The lumen of the main renal artery was displayed. The main renal artery was fully displayed. We obtained the peak systolic velocity (PSV) and resistive index at the stenosed segment of the main renal artery.

According to related literature reports and our own experience, the diagnosis and grading renal artery stenosis by DUS examination was determined by the following criteria [6, 28, 29]: (1) A PSV of 80-200 cm/s indicates a stenosis of grade I (stenosis rate: 30%-49%). (2) A PSV of 200-395 cm/s indicates a stenosis of grade II (stenosis rate: 50%-69%). (3) A PSV of >395 cm/s indicates a stenosis of grade III (stenosis rate: 70%-99%). (4) A PSV of 0 cm/s in a blocked renal artery indicates a stenosis of grade IV (stenosis rate: 100%).

### 2.3. CEUS Examinations

All of the patients took the supine position or lateral position. They kept fasting for more than 6 hours. The long axis section of main renal artery was shown. The lumen of the main renal artery was displayed. SonoVue was selected as the contrast agent which was produced by Bracco Company of Italy. SonoVue was dissolved in 5 ml saline. Then, we switched on the CEUS mode of the ultrasonic equipment. A 1.5 ml SonoVue was injected in 5 s through the elbow vein before the CEUS examination. After the injection of SonoVue, the tube was flushed with 5 ml saline immediately. Continuously, dynamic images were stored in real time for 3 minutes. We examined whether there was a local or diffuse stenosis of the main renal artery. Range of perfusion defects in the main renal artery was observed. The minimum diameter at the stenosed region of the renal artery, and the maximum diameter of the normal renal artery were measured depending on the contrast-enhanced images. The stenosis rate was calculated according to the following equation: stenosis rate = [1 − (*X*/*R*)] × 100%, where *X* is the minimum diameter at the stenosed region of the renal artery, and *R* is the maximum diameter of the normal renal artery. The stenosed main renal arteries were graded into I to IV according to the stenosis rate. The classification criteria were grade I (stenosis rate: 30%-49%), grade II (stenosis rate: 50%-69%), grade III (stenosis rate: 70%-99%), and grade IV (stenosis rate: 100%).

### 2.4. Statistical Analysis

SPSS 24.0 software was used for statistical analysis. The counting data were expressed as the number or percentage of cases, and the measurement data were expressed as mean ± standard deviation. The results of DSA for the renal artery were used as the gold standard. The sensitivity, specificity, accuracy, positive predictive value, and negative predictive value of DUS and CEUS in the diagnosis of renal artery stenosis were calculated. The *X*^2^-test was used to compare the rates. Kappa consistency analysis was performed between ultrasonic examination (DUS or CEUS) and DSA. Data interpretation was classified as follows: poor consistency (kappa = 0.0-0.20), fair consistency (kappa = 0.21-0.40), moderate consistency (kappa = 0.41-0.60), good consistency (kappa = 0.61-0.80), and excellent consistency (kappa = 0.81-1.0) [30]. The ROC curves were plotted. The accuracy of DUS or CEUS in grading renal artery stenosis was evaluated by the area under the receiver operating characteristic curves and compared between groups. *P* < 0.05 was considered statistically significant.

## 3. Results

### 3.1. DSA Results

All of the 122 renal arteries were examined by DSA. Among them, 41 renal arteries had no obvious stenosis. 81 renal arteries had stenosis. And 25 of the 81 renal arteries were classified as grade I, 36 grade II, 16 grade III, and 4 grade IV (Tables 1 and 2).

### 3.2. DUS Results

**Figure 4:**
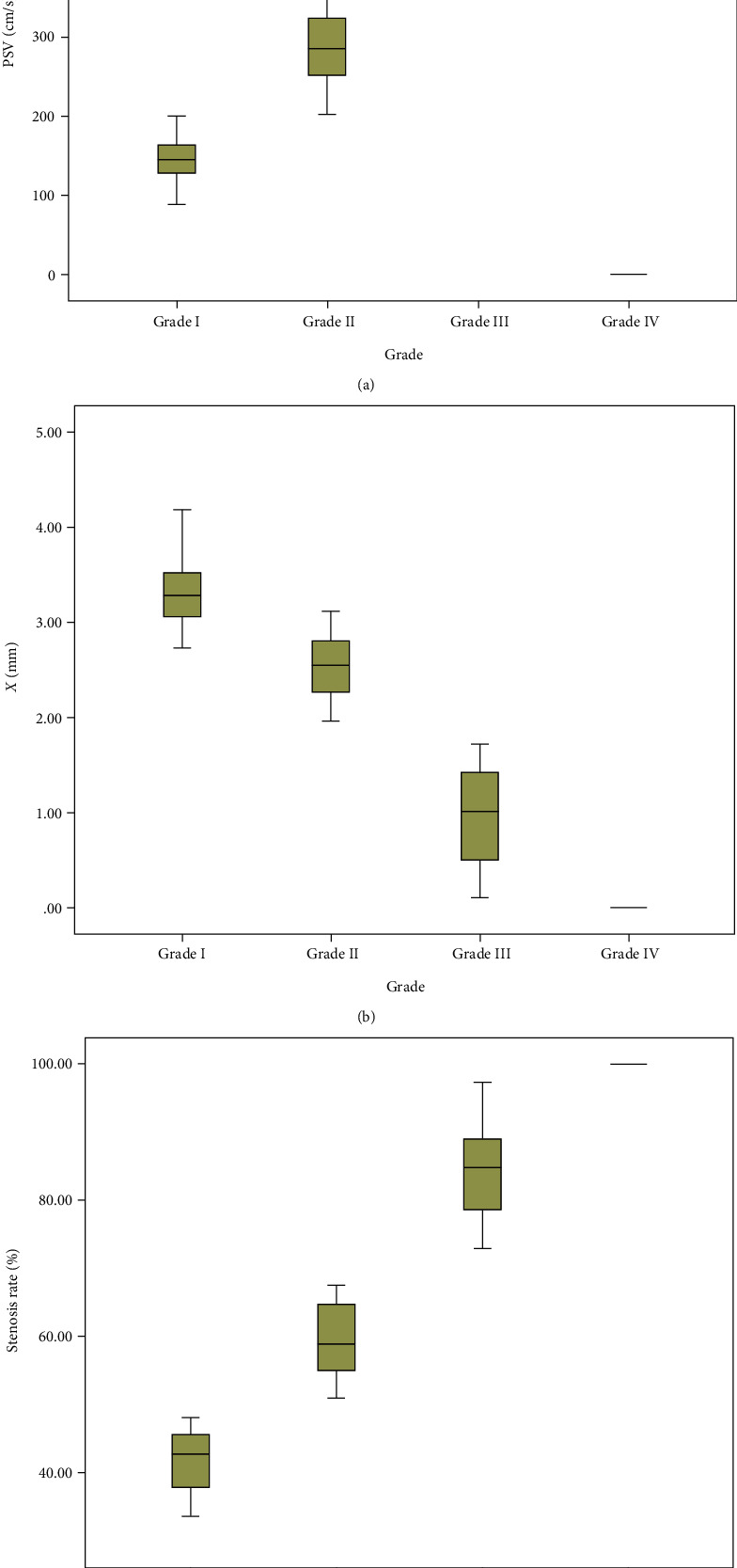
Parameters of DUS and CEUS in the diagnosis and grading renal artery stenosis. (a) PSV parameters of the Doppler ultrasound in the diagnosis and grading renal artery stenosis. (b) *X* parameters of contrast-enhanced ultrasound in the diagnosis and grading renal artery stenosis. (c) Stenosis rate parameters of contrast-enhanced ultrasound in the diagnosis and grading renal artery stenosis. PSV: peak systolic velocity at the stenosed segment of the renal artery by the Doppler ultrasound; *X*: the minimum diameter at the stenosed segment of the renal artery by contrast-enhanced ultrasound.

The 122 renal arteries which were included in the study were examined by the Doppler ultrasound. The main renal artery presented segmental or diffuse stenosis. Among the 81 stenosed renal arteries confirmed by DSA, 60 were diagnosed by DUS, and the other 21 were not detected by DUS (Tables 1 and 2). About 71.2% of the main renal artery stenosis occurred in the proximal end near the abdominal aorta. 5.9% renal artery stenosis was caused by diffuse thickening of the main renal artery. 22.9% renal artery stenosis was found in the middle and distal segments. PSV parameters were obtained at the stenosed segment of the main renal artery by DUS examination (Table 3 and Figure 4(a)). The peak systolic velocity at the stenosed segment of the main renal artery increased from grade I to III. No obvious Doppler signal was showed in the occluded renal artery, and the peak systolic velocity of grade IV was 0 cm/s (Figure 4(a)). And DUS also revealed stenosis in 69 renal arteries. Varying degrees of renal artery stenosis were presented. Of the 69 renal arteries, 19 were classified as grade I, 31 grade II, 16 grade III, and 3 grade IV. There was a significant difference in grading renal artery stenosis between DUS and DSA (*X*^2^ = 4.033, *P* = 0.043) (Tables 1 and 2). The sensitivity, specificity, accuracy, positive predictive value, and negative predictive value of DUS in the diagnosis of renal artery stenosis were 74.1%, 78.0%, 75.4%, 87.0%, and 60.4%, respectively (Table 4).

### 3.3. CEUS Results

Contrast agent was injected through the elbow vein. In patients without renal artery stenosis, the contrast agent was well filled. Among the 81 stenosed renal arteries confirmed by DSA, 72 were diagnosed by CEUS, and the other 9 were not detected by CEUS (Tables 1 and 2). The minimum diameter *X* at the stenosed segment of the renal artery (Table 3 and Figure 4(b)) and the maximum diameter *R* of the normal renal artery were obtained by CEUS examination (Table 3). The renal artery stenosis rate was calculated using the diameters of *X* and *R* according to the following equation: stenosis rate = [1 − (*X*/*R*)] × 100% (Table 3 and Figure 4(c)). The diameter of the main renal artery decreased significantly at the stenosed segment in patients with renal artery stenosis. And the contrast agent could not be fully filled at the stenosed segment of the renal artery. There was no exact contrast agent filled in the occlusive segment of the main renal artery. Among the 122 renal arteries included in the study, CEUS diagnosed 77 renal arteries with different degree of stenosis, including 21 grade I stenosis, 34 grade II stenosis, 19 grade III stenosis, and 3 grade IV stenosis. There was no significant difference in grading renal artery stenosis between CEUS and DSA (*X*^2^ = 0.643, *P* = 0.424) (Tables 1 and 2). The sensitivity, specificity, accuracy, positive predictive value, and negative predictive value of CEUS in the diagnosis of renal artery stenosis were 88.9%, 87.8%, 88.5%, 93.5%, and 80.0%, respectively (Table 4).

### 3.4. Consistency Analysis

There was a moderate consistency between DUS and DSA (kappa = 0.486, *P* < 0.05), which showed that DUS was moderately reliable in the diagnosis of renal artery stenosis. There was a good consistency between CEUS and DSA (kappa = 0.749, *P* < 0.05), which showed that CEUS was reliable in the diagnosis of renal artery stenosis. The consistency analysis showed that CEUS presented a significant advantage over DUS (*P* < 0.05). CEUS is more reliable than the Doppler ultrasound.

**Figure 5:**
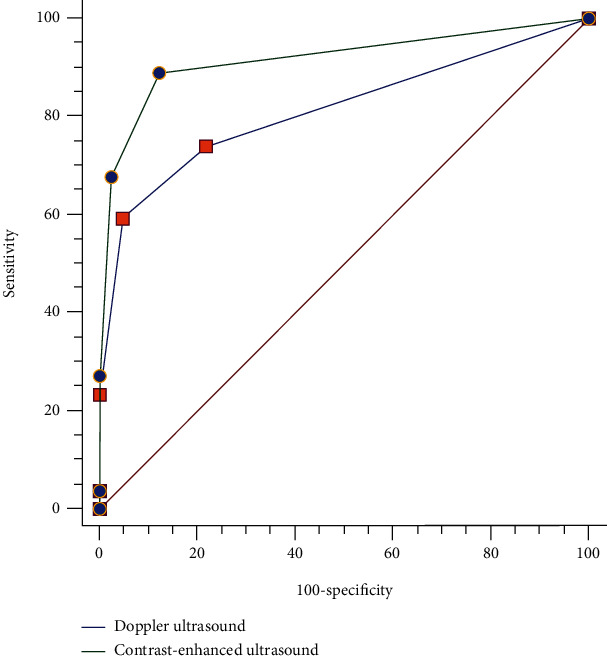
Comparison between the Doppler ultrasound and contrast-enhanced ultrasound in grading renal artery stenosis (*Z* = 3.148, *P* = 0.002).

The area under the ROC curve of DUS in grading renal artery stenosis was 0.813 (95% CI 0.733-0.878). And the area under the ROC curve of CEUS was 0.917 (95% CI 0.854-0.960) (Figure 5). There was a significant difference in grading renal artery stenosis between the two examinations (*Z* = 3.148, *P* = 0.002), indicating that CEUS was superior to DUS in grading renal artery stenosis.

## 4. Discussion

Renal artery stenosis may cause a series of serious complications [31]. Early diagnosis of renal artery stenosis may greatly reduce the risk for complications. Imaging strategies is crucial for patients with renovascular disease. Correct application of imaging techniques for the clinical situation may maximize the diagnostic accuracy, and it could also limit radiation dose and reduce potential adverse events [32]. DUS has been the first-line imaging modality for the diagnosis of renal artery stenosis [33, 34]. It permits the grading of renal artery stenosis by using hemodynamic measurement parameters [35]. However, the results of DUS in different studies are to some extent conflicting [24, 35]. Advanced imaging technique such as CEUS has attracted the attention of researchers [17, 24, 25]. In this current study, renal artery stenosis measurements between CEUS and other imaging modalities were compared. Our results showed that there was a good consistency between CEUS and DSA and a moderate consistency between DUS and DSA in the diagnosis of renal artery stenosis. The accuracy of CEUS in grading renal artery stenosis was superior to DUS. CEUS is a promising method for the diagnosis of renal artery stenosis.

A previous study also used CEUS for the diagnosis of renal artery stenosis and showed good results. They found that the sensitivity, specificity, and accuracy of CEUS were similar to selective angiography [23]. Our study investigated the diagnostic value of CEUS in renal artery stenosis using DSA as the gold standard which is superior to selective angiography that was used in the previous study. The other innovation in our study is that the stenosed renal arteries were classified into four grades according to the degree of stenosis. The diagnosis of different grades of renal artery stenosis using CEUS has not been reported to our knowledge. Similar to previous findings [23], we found that CEUS could increase the feasibility of evaluating renal artery stenosis and it is more reliable than DUS in the diagnosis of renal artery stenosis. The sensitivity, specificity, accuracy, positive predictive value, and negative predictive value of CEUS were higher than that of DUS. The kappa value of consistency test between CEUS and DSA was higher (kappa value = 0.798, *P* < 0.05) than that of DUS (kappa value = 0.448, *P* < 0.05). CEUS is highly consistent with the results of DSA. In our study, the area under the ROC curve was used for the evaluation of grading renal artery stenosis. The area under the ROC curve of CEUS was (0.917, 95% CI 0.854-0.960), which was higher than that of DUS (0.813, 95% CI 0.733-0.878). CEUS was superior to DUS in grading renal artery stenosis. However, we should also note that renal artery stenosis is not always detected by CEUS. In this current study, 9 of the 81 stenosed renal arteries confirmed by DSA were not detected by CEUS. As is known to us, CEUS is technically demanding and operator dependent. CEUS can also be affected by obesity, depth, bowel gas, complex anatomy, etc., which may lead to an inadequate visualization of the renal artery in DUS. On equal terms, CEUS can greatly overcome the deficiency of adverse acoustic window conditions and enhance the display of renal arteries. Professionals experienced in CEUS techniques are required to perform the examinations. A low-frequency probe is necessary for the localization of the renal arteries especially for obese individuals. And the localization of renal artery from the upper abdominal aorta in the cross-section and dosing pressure with a probe may reduce the required depth of penetration and suppress artifacts produced by bowel gas. CEUS was generally performed in the early morning after an overnight fast to minimize bowel gas. Doppler parameters such as the PSV, peak velocity ratio, resistive index, and systolic acceleration time were routinely used for evaluating the degree of stenosis [35]. But there is no consensus on the best Doppler parameter to evaluate renal artery stenosis as each parameter has its advantages and disadvantages. Resistive index and systolic acceleration time only offer reliable accuracy in diagnosing of high-grade stenosis. PSV is the most commonly used parameter, but it can also be influenced to some extent due to angle measurement errors, arterial wall rigidity, and chronic renal disease [36]. It is difficult to reliably detect renal artery stenosis using the Doppler ultrasound. CEUS can significantly improve the clarity of renal artery blood flow. Grading renal artery stenosis can be effectively determined by the subtraction of diameter through CEUS examinations.

This article has two limitations: (1) Although there is a good consistency between CEUS and DSA, the accuracy of CEUS still need to be improved, especially in patients with overlying fat or bowel gas. (2) Although the reason of renal artery stenosis was not related to the purpose of our study, different reasons may lead to different management strategies in spite of the same degree of stenosis. The other study may be needed to assess this aspect.

## 5. Conclusion

CEUS can enhance the flow visualization of renal artery stenosis, and it showed a good consistency with the gold standard DSA. As a noninvasive, nonradiative, nontoxic, accurate, and cost-effective technique, CEUS may represent the method of choice in grading renal artery stenosis.

## Figures and Tables

**Table 1:** Diagnostic value of DUS and CEUS in renal artery stenosis.

	DSA	DUS		CEUS	
		+	-	+	-
+	81	60	21	72	9
-	41	9	32	5	36
Total	122	69	53	77	45
P		0.043∗		0.424∗∗	

DSA: digital subtraction angiography; CEUS: contrast-enhanced ultrasound; DUS: Doppler ultrasound. ^∗^There was a significant difference in grading renal artery stenosis between DUS and DSA (*P* = 0.043). ^∗∗^There was no significant difference in grading renal artery stenosis between CEUS and DSA (*P* = 0.424).

**Table 2:** Number of renal artery stenosis classified as different grades.

	DSA	DUS	CEUS
Grade I	25	19	21
Grade II	36	31	34
Grade III	16	16	19
Grade IV	4	3	3

Data are reported as count. DSA: digital subtraction angiography; CEUS: contrast-enhanced ultrasound; DUS: Doppler ultrasound; grade I (stenosis rate: 30%-49%), grade II (stenosis rate: 50%-69%), grade III (stenosis rate: 70%-99%), and grade IV (stenosis rate: 100%).

**Table 3:** Parameters of DUS and CEUS in the diagnosis and grading renal artery stenosis.

	SR (%), median (min, max)	PSV (cm/s)	*X* (mm)	*R* (mm)
Grade I	42.76 (33.65, 48.12)	134.97 ± 37.86	3.60 ± 0.58	5.82 ± 0.79
Grade II	58.89 (51.01, 67.55)	283.45 ± 56.44^∗^	2.43 ± 0.47^∗^	5.75 ± 0.84
Grade III	84.81 (72.94, 97.27)	467.31 ± 61.72^∗^^#^	1.03 ± 0.49^∗^^#^	5.79 ± 0.43
Grade IV	100	0	0	5.68 ± 0.77
P	—	<0.001	<0.001	0.976

Grade I (stenosis rate: 30%-49%), grade II (stenosis rate: 50%-69%), grade III (stenosis rate: 70%-99%), and grade IV (stenosis rate: 100%); SR: stenosis rate; PSV: peak systolic velocity at the stenosed segment of the renal artery by the Doppler ultrasound; *X*: the minimum diameter at the stenosed segment of the renal artery by contrast-enhanced ultrasound; *R*: maximum diameter of the normal renal artery by contrast-enhanced ultrasound.

**Table 4:** Diagnostic value of CEUS and DUS in the diagnosis of renal artery stenosis.

	Sensitivity	Specificity	Accuracy	PPV	NPV
DUS	74.1%	78.0%	75.4%	87.0%	60.4%
CEUS	88.9%	87.8%	88.5%	93.5%	80.0%

PPV: positive predictive value; NPV: negative predictive value.

## Data Availability

The data generated during the study are available from the corresponding author upon reasonable request.
